# Emerging Therapies for Familial Lecithin-Cholesterol Acyltransferase Deficiency: A Role for Plasma Exchange

**DOI:** 10.1016/j.ekir.2024.04.026

**Published:** 2024-04-16

**Authors:** Aruni Ratnayake, Marta Turri, Laura Calabresi, Chiara Pavanello, Adam McLean, Anisha Tanna, Jaimini Cegla, Ben Jones, Neill Duncan

**Affiliations:** 1 Imperial College Renal and Transplant Center, Imperial College Healthcare NHS Trust, Hammersmith Hospital, London, UK; 2 Centro Grossi Paoletti, Dipartimento di Scienze Farmacologiche Biomolecolari, Università degli Studi di Milano, Italy; 3 Department of Lipids and Cardiovascular Risk Service, Imperial College Healthcare NHS Trust, Hammersmith Hospital, London, UK; 4 Section of Endocrinology and Investigative Medicine, Department of Metabolism, Digestion and Reproduction, Imperial College London, London UK

## Introduction

Familial lecithin-cholesterol acyltransferase (LCAT) deficiency (FLD) is a rare disorder of lipid metabolism causing decreased maturation of high density lipoprotein particles and impaired cholesterol esterification.^1^ Patients with FLD develop corneal opacification, hemolytic anemia, proteinuria, and lipid abnormalities including elevated triglycerides, reduced high density lipoprotein cholesterol, and increased unesterified cholesterol rich lipoprotein X (LpX).^1^ Renal failure is a major cause of mortality.S1 Experimental evidence from mouse models has shown LpX is associated with progressive renal disease.^2^ Patients who develop end-stage renal failure are candidates for renal transplantation. However, the disease can recur in transplanted grafts.^3^^,^S2 There is no curative treatment; management is focused on correction of lipid profile and renoprotective agents.S3

We report a patient with FLD whose renal dysfunction progressed to chronic kidney disease stage 5. We outline the novel use of plasma exchange (PLEX) to correct metabolic defects. PLEX has been shown to lower LpX levels,^4^ thereby reducing accumulation in kidney tissues. In addition, we describe options considered regarding renal replacement therapy.

## Case Presentation

Our patient is a 44-year-old Caucasian male diagnosed with LCAT deficiency in his late teens. A complete lipid profile was determined using a Roche Integra c311 analyzer as previously described.^5^ Cholesterol esterification rate (CER) and LCAT activity were tested by previously described in-house assays.S4 These showed no CER or LCAT activity, confirming FLD phenotype. Sanger sequencing identified the patient was heterozygous for c.560T>C p.(Leu187Pro) and c.809C>T p.(Thr270lle) variants of uncertain clinical significance in the LCAT gene.

Proteinuria developed from 2004, and renal biopsy confirmed histological features of LCAT deficiency (Supplementary Figure S1). Baseline creatinine was 100 to 120 μmol/l, and urine protein-to-creatinine ratio was 150 mg/mmol. By 2019, renal function worsened (creatinine 214 μmol/l; urine protein-to-creatinine ratio 481 mg/mmol). Repeat renal biopsy was performed, showing 50% interstitial fibrosis and tubular atrophy (compared to 10% in the initial biopsy). It was explained that preparations would need to be made regarding renal replacement therapy. The patient was keen to explore novel treatment options of LCAT deficiency while initiating transplant work-up investigations concurrently.

**Figure 1:**
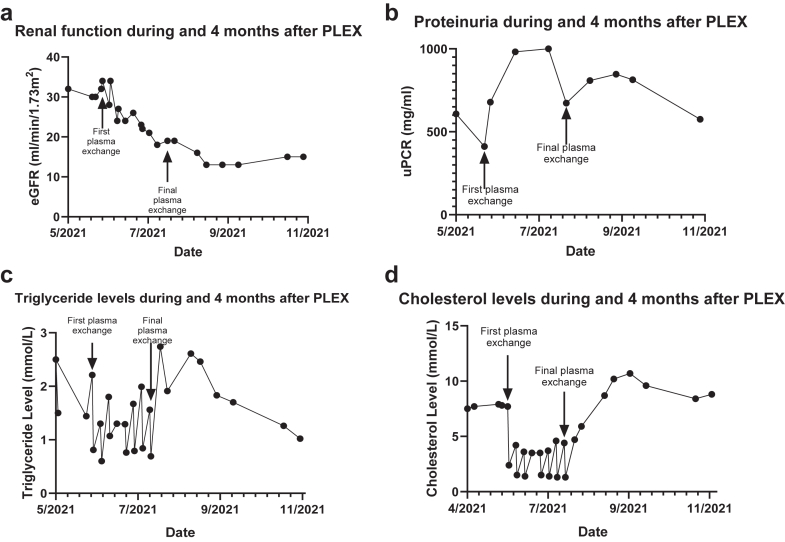
Graphs showing trends in (a) renal function, (b) proteinuria, (c) triglyceride, and (d) cholesterol levels during and after plasma exchange. PLEX, plasma exchange.

Therefore, PLEX therapy was trialed. A cuffed central venous catheter (Tesio, Medcomp) was inserted, and the patient commenced weekly PLEX for 8 weeks with 5% albumin solution, which was well-tolerated. Unfortunately, renal function deteriorated further, and urine protein-to-creatinine ratio increased (Figure 1a and b). Although plasma volume shifts during PLEX may have caused acute tubular necrosis, the increased proteinuria was not explained. PLEX was subsequently discontinued.

Blood samples pre-PLEX and post-PLEX showed that treatment reduced triglycerides and total cholesterol levels, but with notable rebound between sessions. Levels returned to baseline when PLEX was stopped (Figure 1c and d).

**Figure 2:**
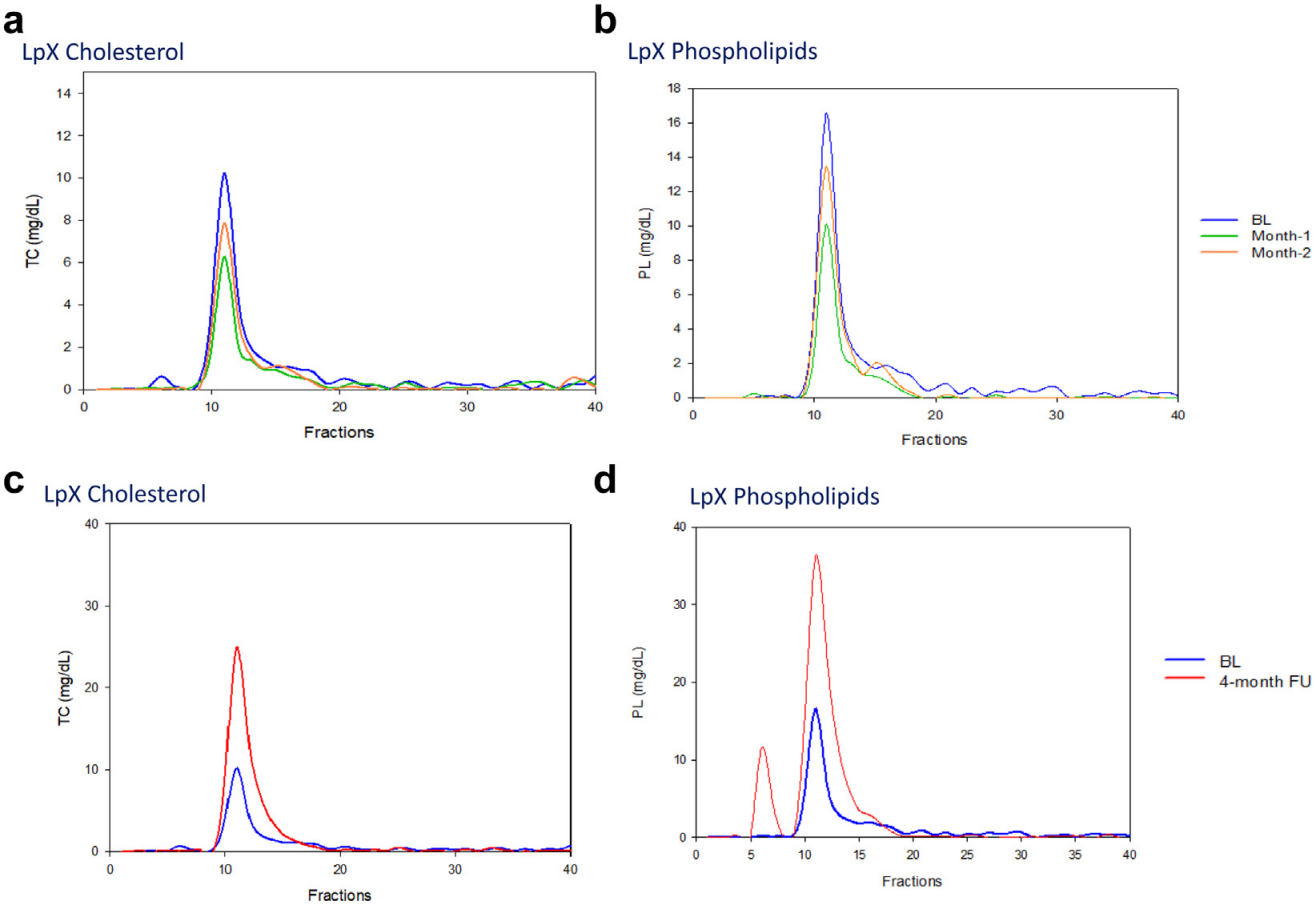
Fast Protein Liquid Chromatography (FPLC) of 1.020 to 1.063 g/ml plasma fraction at baseline, with PLEX treatment (a and b), and 4 months after PLEX cessation (c and d).

After 1 month of treatment, plasma lipids diminished and unesterified-to-total cholesterol ratio decreased by 11.9% (data not shown). Plasma samples were analyzed for LpX fraction, isolated by ultracentrifugation followed by fast protein liquid chromatography. The decrease in phospholipids and cholesterol in the LpX fraction compared to baseline, suggests remodeling of lipoprotein profile and partial removal of LpX (Figure 2a and b). There was subsequent return of elevated LpX 4 months after PLEX cessation (Figure 2c and d).

With declining renal function, pre-emptive kidney transplantation was considered. The patient has 2 sisters: one with LCAT deficiency, the other asymptomatic. His unaffected sister came forward as a potential live donor. She had no stigmata of FLD, normal renal function, and no proteinuria. However, preliminary health screening found she carried the same LCAT variant (c.560T>C) as her brother, negating her ability to be a donor. Currently, he is listed for deceased donor kidney transplantation, and he commenced hemodialysis in the interim.

## Discussion

This case demonstrates the first time PLEX has been used therapeutically in FLD. There are case reports highlighting benefit of PLEX for hypertriglyceridemia causing therapeutic reduction of LpX with minimal adverse effects.^4^^,^S5–S7 PLEX reduced LpX levels in our patient, which was not maintained following cessation and is not a sustainable long-term intervention. Moreover, kidney function deteriorated further after PLEX was commenced. Of note, our patient had advanced kidney disease and renal scarring; it is unknown whether PLEX-mediated lowering of LpX may have slowed rate of renal decline if treatment had commenced at an earlier stage of kidney disease.

There has been increased interest in the high density lipoprotein mimetic peptide CER-001 (Abionyx). There was a case report of successful administration of CER-001 in patients with LCAT deficiency, which reduced LpX levels, and halted decline in renal function and proteinuria.^6^ Conversely, another case report of administration of CER-001 in LCAT deficiency showed reduction of LpX levels but no significant impact on renal function in a patient with aggressive LCAT disease on their third renal transplant with baseline estimated glomerular filtration rate <20 ml/min.^5^

Furthermore, there was an *n* = 1 trial implanting genetically modified adipocytes expressing LCAT.^7^ Initial improvement in proteinuria was observed with no severe adverse events reported. Our patient was very keen to explore this potentially curative option. Due to limited data with unclear long-term outcomes, this option was not considered.

Kidney transplantation is the optimal form of renal replacement therapy.S8 However, disease recurrence affects longevity of kidney transplants.^3^^,^S2 The best outcome might be with combined liver-kidney transplantation; transplantation of the liver, where LCAT is predominantly synthesized,S9 would correct the underlying metabolic defect. There have only been 2 case reports of liver-kidney transplantation for LCAT deficiency.^8^^,^^9^ Ahmad *et al.*^8^ reported a case of sequential kidney-liver transplantation from the same living donor. At 5 years follow-up, the patient had good liver and kidney transplant function with no histological evidence of disease recurrence or rejection. Alfaro *et al.*^9^ also reported a case of sequential kidney-liver transplantation, where there was no evidence of vascular compromise or rejection 4 years posttransplant. However, in this case renal function progressively declined, leading to a second kidney transplant 10 years after the first.

For our patient, the hepatology transplant team advised whole liver transplantation to provide sufficient levels of LCAT production. However, the patient did not wish to follow this approach of more invasive surgery based on only 2 published case reports.^8^^,^^9^ He preferred to be listed for renal transplant alone pending potential future development of LCAT therapies.

### Patient Perspective

Our patient has been central to the management of his case. He was kept informed of all treatment options and was willing to undergo PLEX therapy with full transparency this had never been trialed in LCAT deficiency.

He has read the literature extensively and is particularly interested in receiving post–renal transplant small molecule therapy with CER-001^6^ or gene therapy.^7^ Given that LCAT deficiency is rare, the number of patients included in trials are very low, thereby reducing the likelihood that effective pharmacological or genetic treatment will be developed in the immediate foreseeable future.

## Conclusion

This is the first reported case assessing PLEX therapy in LCAT deficiency. We have demonstrated that PLEX reduces nephrotoxic LpX levels. However, in our case PLEX was associated with worsening renal function on a background of advanced kidney disease with significant scarring. This suggests potential unknown side effects to renal function of PLEX beyond nephrotoxic effects of LpX. Timing of PLEX administration, however, could influence disease progression because it could potentially be used at an earlier stage of kidney disease to slow rate of renal decline. Further investigations are required to evaluate long-term effects on renal outcomes in this rare condition with limited treatment options (Table 1).

**Table 1:** Teaching points

PLEX reduces levels of nephrotoxic LpX levels in lecithin-cholesterol acyltransferase deficiency, with minimal symptomatic side effects.
Reduction of LpX levels has therapeutic potential to slow the rate of decline of kidney function in patients without significant renal tissue scarring.
PLEX does not correct the underlying metabolic defect; rebound increase of LpX and triglycerides raises logistical queries of feasibility of plasma exchange as a long-term therapeutic option.
Involvement of the multidisciplinary team and detailed patient communication is important in this rare condition with limited treatment options.

LpX, lipoprotein X; PLEX, plasma exchange.

## Disclosure

ND is a member of the Advisory Board for Fresenius Medical Care and Astra Zeneca. All the other authors declared no competing interests.

## Patient Consent

The authors declare that they have obtained consent from the patient discussed in the report.

## Supplementary Material

Supplementary Material (PDF)

Supplementary Reference.

Figure S1. Renal biopsy results from patient taken in 2019. (a) Light microscopy showing enlarged glomerulus with variable expansion of mesangial matrix which has a spongy appearance, characteristic of LCAT deficiency (200x). (b) Silver stain light microscopy highlighting capillary walls with complex vacuolated appearance, and dilated capillary lumens with foamy material within the lumen (200x). (c) Electron microscopy showing irregularly thickened glomerular basement membrane and intramembranous and subendothelial electron-dense, rounded lamellar bodies. There are regions of extensive foot process effacement of epithelial cells and some surface microvilli are present. Some mitochondria in proximal tubules are enlarged and have distorted cristae. Immunofluorescence (not shown) identified in one glomerulus: mesangial IgM+, Segmental IgM +++, C3c+++, C1q+ positive. Serum complement levels were normal.

## Footnotes

Supplementary Material (PDF)

**Supplementary References.**
**Figure S1.** Renal biopsy results from patient taken in 2019. (a) Light microscopy showing enlarged glomerulus with variable expansion of mesangial matrix which has a spongy appearance, characteristic of LCAT deficiency (200x). (b) Silver stain light microscopy highlighting capillary walls with complex vacuolated appearance, and dilated capillary lumens with foamy material within the lumen (200x). (c) Electron microscopy showing irregularly thickened glomerular basement membrane and intramembranous and subendothelial electron-dense, rounded lamellar bodies. There are regions of extensive foot process effacement of epithelial cells and some surface microvilli are present. Some mitochondria in proximal tubules are enlarged and have distorted cristae. Immunofluorescence (not shown) identified in one glomerulus: mesangial IgM+, Segmental IgM +++, C3c+++, C1q+ positive. Serum complement levels were normal.
