# Supplementary material for: Emerging Therapies for Familial Lecithin-Cholesterol Acyltransferase Deficiency: A Role for Plasma Exchange
Source: Kidney Int Rep. 2024 Apr 16;9(7):2299–302. doi: 10.1016/j.ekir.2024.04.026 (PMC11284401; doi:10.1016/j.ekir.2024.04.026)
Supplement: Supplementary Material (PDF) [file mmc1.pdf]

SUPPLEMENTAL MATERIAL

Supplementary Figure S1 Legend

Renal biopsy results from patient taken in 2019. (a) Light microscopy showing enlarged glomerulus with variable expansion of mesangial matrix which has a spongy appearance, characteristic of LCAT deficiency (200x). (b) Silver stain light microscopy highlighting capillary walls with complex vacuolated appearance, and dilated capillary lumens with foamy material within the lumen (200x). (c) Electron microscopy showing irregularly thickened glomerular basement membrane and intramembranous and subendothelial electron-dense, rounded lamellar bodies. There are regions of extensive foot process effacement of epithelial cells and some surface microvilli are present. Some mitochondria in proximal tubules are enlarged and have distorted cristae. Immunofluorescence (not shown) identified in one glomerulus: mesangial IgM+, Segmental IgM +++, C3c+++, C1q+ positive. Serum complement levels were normal.

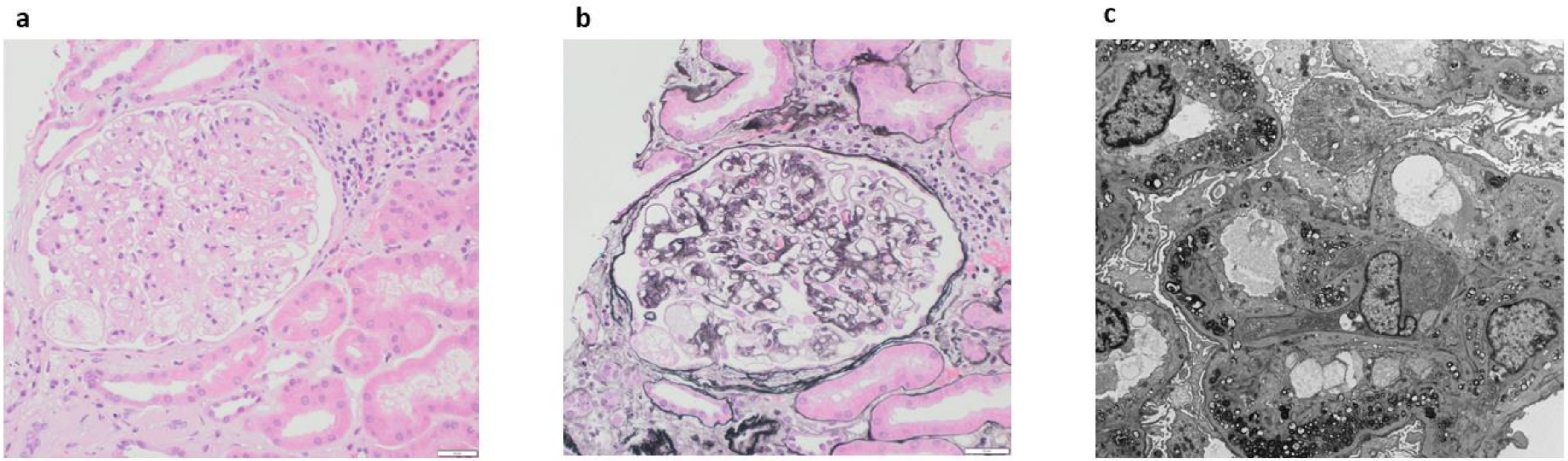
a
b
c

Supplementary References

S1 Morales E, Alonso M, Sarmiento B et al. LCAT deficiency as a cause of proteinuria and corneal opacification. *BMJ Case Rep*. 2018;2018:bcr2017224129. Published 2018 Mar 13. doi:10.1136/bcr-2017-224129

S2 Strøm EH, Sund S, Reier-Nilsen M, et al. Lecithin: Cholesterol Acyltransferase (LCAT) Deficiency: renal lesions with early graft recurrence. *Ultrastruct Pathol*. 2011;35(3):139-145. doi:10.3109/01913123.2010.551578

S3 Aranda P, Valdivielso P, Pisciotta L, et al. Therapeutic management of a new case of LCAT deficiency with a multifactorial long-term approach based on high doses of angiotensin II receptor blockers (ARBs). *Clin Nephrol*. 2008;69(3):213-218. doi:10.5414/cnp69213

S4 Pavanello C, Ossoli A, Arca M, et al. Progression of chronic kidney disease in familial LCAT deficiency: a follow-up of the Italian cohort. *J Lipid Res*. 2020;61(12):1784-1788. doi:10.1194/jlr.P120000976

S5 Kim AS, Hakeem R, Abdullah A, et al. Therapeutic plasma exchange for the management of severe gestational hypertriglyceridaemic pancreatitis due to lipoprotein lipase mutation. *Endocrinol Diabetes Metab Case Rep*. Published online March 13, 2020. doi:10.1530/EDM-19-0165

S6 Santos A, Ferreira F, Brás C, et al. Plasmapheresis as an Alternative Treatment of Hypertriglyceridemia-Induced Pancreatitis: A Case Report. *Cureus*. 2022;14(11):e32000. Published 2022 Nov 29. doi:10.7759/cureus.32000

S7 Ramírez-Bueno A, Salazar-Ramírez C, Cota-Delgado F et al. Plasmapheresis as treatment for hyperlipidemic pancreatitis. *Eur J Intern Med*. 2014;25(2):160-163. doi:10.1016/j.ejim.2013.08.701

S8 Tonelli M, Wiebe N, Knoll G, et al. Systematic review: kidney transplantation compared with dialysis in clinically relevant outcomes. *Am J Transplant*. 2011;11(10):2093-2109. doi:10.1111/j.1600-6143.2011.03686.x

S9 Rousset X, Shamburek R, Vaisman B et al. Lecithin cholesterol acyltransferase: an anti- or pro-atherogenic factor? *Curr Atheroscler Rep*. 2011;13(3):249-256. doi:10.1007/s11883-011-0171-6
